# Effect of dexamethasone intravitreal implant (Ozurdex®) on corneal endothelium in retinal vein occlusion patients: Corneal endothelium after dexamethasone implant injection

**DOI:** 10.1186/s12886-018-0905-0

**Published:** 2018-09-04

**Authors:** Hatice Ayhan Güler, Nurgül Örnek, Kemal Örnek, Nesrin Büyüktortop Gökçınar, Tevfik Oğurel, Mehmet Erhan Yumuşak, Zafer Onaran

**Affiliations:** 1 Department of Ophthalmology, Faculty of Medicine, Kırıkkale University, Kırıkkale, Turkey; 2 Department of Ophthalmology, Kudret Eye Hospital, Ankara, Turkey; 3 Department of Ophthalmology, Bayburt State Hospital, Bayburt, Turkey

**Keywords:** Dexamethasone implant, Retinal vein occlusion, Corneal endothelium, Specular microscopy

## Abstract

**Background:**

To assess corneal endothelial cell changes after intravitreal dexamethasone (DEX) implant (Ozurdex®) injection in patients with macular edema secondary to retinal vein occlusion (RVO).

**Methods:**

Twenty-two eyes of 22 patients were assessed prospectively after intravitreal 0.7 mg DEX implant injection. Twenty-two eyes of 22 healthy volunteers served as control group. Corneal endothelial cell parameters including endothelial cell density (ECD), coefficient of variation of cell size (CV), percentage of hexagonality (Hex) and central corneal thickness (CCT) were analyzed before and 1 and 3 months after injection by specular microscopy. The results of the study were compared statistically.

**Results:**

There were 17 (77.3%) patients with branch RVO and 5 (22.7%) patients with central RVO. Mean intraocular pressure (IOP) was 14.73 mmHg before injection, 17.05 mmHg at 1 month and 17.15 mmHg at 3 months after injection. Mean IOP at 1 and 3 months were significantly higher than pre-injection value (*p* = 0.002 and *p* = 0.003, respectively). There was a statistically significant reduction in mean ECD at 3 months after injection compared to pre-injection and 1 month (*p* = 0.013, *p* = 0.009, respectively) in the injected eyes. Mean ECD showed no significant difference in the uninjected fellow eyes during the follow up (p>0.05). Mean CV and Hex did not reveal a statistically significant difference in injected and uninjected fellow eyes (*p* > 0.05). No significant change was observed in mean CCT values during the follow up (*p* = 0.8).

**Conclusion:**

Intravitreal dexamethasone implant may cause a transient reduction in corneal endothelial cell density in short term without changing cell morphology.

## Background

Corticosteroids are widely used in ophthalmology for their anti-inflammatory, antipermeability and antifibrotic properties. They modulate cellular proliferation, apoptosis, and development. Steroids suppress inflammation by immobilizing arachidonic acid, downregulating multiple cytokine pathways including vascular endothelial growth factor (VEGF) pathway, stabilizing cell membranes and mast cell granules, inhibiting leukocyte interaction and slowing diapedesis.

Dexamethasone is a type of synthetic corticosteroid. It is one of the most commonly used corticosteroid in ophthalmology with similar indications as other corticosteroid preparations. Anti-inflammatory activity of dexamethasone is about six times stronger than that of prednisone or prednisolone and 30 times that of cortisone.

Ozurdex® is an intravitreal implant containing 700 μg preservative-free dexamethasone (DEX) in a slow release drug delivery system. Because of its anti-inflammatory and anti-angiogenic effect, DEX implant is indicated for various posterior segment diseases, like macular edema due to retinal vein occlusion (RVO), diabetic maculopathy and non-infectious posterior uveitis etc. [1–5].

Glucocorticoid receptors and messenger ribonucleic acids (mRNA) regulating glucocorticoid activity at these receptors were found in corneal endothelium [6, 7]. Effect of DEX implant on corneal endothelium has been studied in very few studies. Kwak et al. reported no toxic effect on cornea, retina and lens in a rabbit model following 400 mg intravitreal DEX injection [8]. İlhan et al. reported that 0.7 mg intravitreal DEX implant application probably have no side effect on corneal endothelium at six months in patients with macular edema caused by RVO [9].

The aim of the study was to evaluate effect of intravitreal dexamethasone implant (Ozurdex®) on corneal endothelium in patients with macular edema secondary to RVO.

## Methods

This prospective clinical study was conducted between September 2015 and September 2016 at the Ophthalmology Department of Kırıkkale University Hospital. It was approved by local ethics committee and was in accordance with the Declaration of Helsinki. The patients were informed before the study and all signed the consent forms.

There were 22 eyes of 22 patients with RVO and macular edema in the study group. Twenty two eyes of 22 healthy volunteers served as controls. Participants who were under 18 years or over 80 years and those who had pregnancy, glaucoma, contact lens use, previous intravitreal injection, ocular trauma, uveitis, endothelial cell count less than 1500 cells/mm^2^ and corneal opacity were excluded.

Complete ophthalmologic examination was performed at each visit including best-corrected visual acuity (BCVA), intraocular pressure (IOP) measurement, biomicroscopy, fundus examination and optical coherence tomography (OCT) imaging. IOP was measured by Goldman applanation tonometry (CSO®, Italy) before and at 1 and 3 months after intravitreal injection. OCT scans of macula were demonstrated using spectral domain OCT (Retinascan Advanced RS-3000, NIDEK, Gamagori, Japan). Fundus fluorescein angiography (Canon CF-1®, Japan) was performed before injection.

Endothelial cell density (ECD), coefficient of variation of cell size (CV), percentage of hexagonality (Hex) were measured from right eyes of volunteers and the injected and uninjected fellow eyes of patients before and at 1 and 3 months after injection using corneal specular microscopy (Konan Noncon Robo SP8000, Konan Medical, Hyogo, Japan). A single examiner evaluated corneal endothelial cell parameters using central analysis method. In this method, at least 110 neighbouring cells were manually marked centrally for endothelial analysis and the imagenet software program displayed the results automatically. Central corneal thickness (CCT) was measured automatically by specular microscopy.

Dexamethasone implant was injected after topical anesthesia by proparacaine hydrochloride and surface disinfection with %5 povidone iodine. Dexamethasone implant was delivered through a 22-gauge needle, with a preloaded applicator and inserted into the vitreous cavity through pars plana. Topical moxifloxacine drop was used for 5 days after injection.

For assessing repeatability of corneal endothelial cell count measurements (ECD, CV, percentage of Hex) same baseline images of 22 injected eyes were analyzed twice on separate days by the same examiner using central analysis method. The difference between continuous variables was tested using one sample t test and repeatability of each pair of analysis was assessed using the 95% limit of agreement (LOA) calculated as mean difference ± 1.96 x SD of the difference according to Bland and Altman. Intraclass correlation coefficient (ICC) was measured to reveal reliability. ICC value should not be less than 0.9 in most clinical measurements.

Statistical analyses were performed using SPSS for Windows 22.0 (SPSS İnc., Chicago, IL). A *p* value below 0.05 was considered statistically significant.

## Results

The study included 5 (22.7%) patients with central retinal vein occlusion (CRVO) and 17 (77.3%) patients with branch retinal vein occlussion (BRVO). Twenty-two eyes of 22 healthy volunteers served as control group. There were 14 females and 8 males. Mean age of the patients was 60.9 (range: 40–75) years. There were 14 phakic and 8 pseudophakic patients. There was no significant difference in terms of gender and age between control and study groups (*p* = 0.678, *p* = 0.940, respectively).

In comparison to control eyes, there was no statistically significant difference in mean ECD, CV, Hex and CCT measurements of injected and uninjected fellow eyes of the study group before injection (all *p* > 0.05) (Table 1).

**Table 1:** ECD, CV, Hex and CCT values of control eyes and patient eyes before intravitreal injection

	Control Eyes	Injected Eyes	Uninjected Eyes	*p* value
ECD	2199.5 ± 325.1	2211.7 ± 370.6	2219.8 ± 263.2	0.608* 0.307**
CV	37.68 ± 5.7	37.86 ± 5.5	40.7 ± 8.5	0.883* 0.082**
Hex	54.5 ± 6.9	54.9 ± 6.8	53.7 ± 8.5	0.871* 0.241**
CCT	566.7 ± 42.4	567.5 ± 43.0	556.9 ± 35.2	0.830* 0.522**

*ECD* Endothelial cell density, *CV* Coefficient of variation of cell size, *Hex* Percentage of hexagonality and *CCT* Central corneal thickness by corneal specular microscopy. * shows statistical difference between control and injected eyes, ^**^ shows statistical difference between control and uninjected fellow eyes

Mean BCVA was 0.99 ± 0.75 logMAR (range: 0.20–2.20) and mean foveal thickness was 462.4 ± 96.1 μm (range: 306–600) before intravitreal dexamethasone implant injection. Argon laser treatment was applied to peripheral retina in 4 patients. Three months after intravitreal DEX implant, mean BCVA was increased to 0.46 ± 0.76 logMAR (range: 0–3.0) (*p* = 0.033) and mean foveal thickness was decreased to 316.41 ± 92.48 μm (range:186–552) (*p* < 0.001).

Mean CCT was measured 567.5 ± 43.0 μm before injection, 564.1 ± 43.9 μm at 1 month and 556.5 ± 44.3 μm at 3 months after intravitreal injection in injected eyes. There was no significant difference between mean CCT values before intravitreal injection and at 1 and 3 months after intravitreal injection (*p* = 0.4, *p* = 0.5, respectively) (Table 2).

**Table 2:** ECD, CV, Hex and CCT values of injected and uninjected eyes before intravitreal injection and follow-up visits

		Before injection	1st month	3rd month	*p* value*
CD	Injected eyes	2211.7 ± 370.6	2207.1 ± 351.9	2163.8 ± 357.7^ab^	0.018
	Uninjected eyes	2219.8 ± 263.2	2265.9 ± 254.2	2102.8 ± 551.9	0.179
CV	Injected eyes	37.86 ± 5.55	40.14 ± 6.47	40.05 ± 5.22	0.511
	Uninjected eyes	40.70 ± 8.46	41.35 ± 5.85	41.94 ± 9.22	0.842
Hex	Injected eyes	54.86 ± 6.84	55.81 ± 7.32	56.63 ± 8.25	0.481
	Uninjected eyes	53.70 ± 8.55	53.95 ± 7.52	53.72 ± 8.92	0.879
CCT	Injected eyes	567.5 ± 43.0	564.1 ± 43.9	556.5 ± 44.3	0.810
	Uninjected eyes	556.9 ± 35.2	558.8 ± 39.7	563.3 ± 41.2	0.104

*Friedman Test, aPost-hoc: Statistical difference detected before intravitreal injection and third month (*p* = 0.013), bPost-hoc: Statistical difference detected between first and third month (*p* = 0.009), *ECD* Endothelial cell density, *CV* Coefficient of variation of cell size, *HEX* Percentage of hexogonality and *CCT* Central corneal thickness by corneal specular microscopy

Mean ECD at 3 months after intravitreal injection was statistically significantly lower compared to pre-injection and 1 month values in injected eyes (*p* = 0.013 and *p* = 0.009, respectively). There was no significant difference in ECD in uninjected fellow eyes of patients during follow up (p>0.05). No significant difference was observed in mean CV, Hex and CCT values between injected and uninjected fellow eyes (all *p* > 0.05) (Table 2).

Mean difference (bias) was 3.77 cells/mm^2^ for ECD, − 0.41 for CV and 0.27% for Hex. One sample t test showed no significant difference between 2 measurements (*p* = 0.120 for ECD, *p* = 0.451 for CV and *p* = 0.718 for Hex). Limit of agreement (LOA) (mean difference ± 1.96 x SD) values were 3.77 ± 21.39 cells/mm^2^, − 0.41 ± 12.26 and 0.27 ± 6.85% for ECD, CV and percentage of Hex respectively. LOA values showed good agreement between two analyses. Intraclass correlation coefficient (ICC) value was measured as 0.99 for ECD, 0.93 for CV and 0.90 for Hex which suggested good reliability of measurements.

Mean IOP was 14.73 ± 3.58 mmHg before injection, 17.05 ± 4.40 mmHg at 1 month and 17.15 ± 6.65 mmHg at 3 months after intravitreal injection. Mean IOP at 1and 3 months after injection were statistically significantly higher than pre- injection value (*p* = 0.002, *p* = 0.003, respectively). Only 4 eyes (%18) had IOP higher than 21 mmHg. All were succesfully treated with anti-glaucomatous drops.

Two eyes (9%) had subconjunctival hemorrhage after intravitreal injection. According to the Lens Opasification Classification System (LOCS) 3 scale, mean cataract grade was increased significantly 3 months after intravitreal injection (*p* = 0.001). Mean LOCS 3 scale was 1.4 ± 0.5 (range:1–2) before intravitreal injection and was increased to 2.3 ± 1.1 (range:1–4) 3 months after intravitreal injection.

## Discussion

Retinal vein occlusion is a common disease of retinal vasculature [10]. Macular edema is a frequent cause of visual loss in RVO patients. There are several methods available for treatment. Laser photocoagulation may decrease macular edema in BRVO patients but typically does not improve visual acuity [11].

Options for treatment of macular edema secondary to RVO have expanded in the past few years. Two types of drugs have emerged as an alternative treatment for macular edema in RVO; corticosteroids and anti-VEGF agents. Intravitreal steroid or anti-VEGF injections have been shown to effectively reduce macular edema and improve visual acuity in BRVO and CRVO patients [12, 13]. Good tolerance was observed for a 12-month period for 0.7 mgDEX implant with significantly lesser adverse effects compared to triamcinolone [14].

Sustained release DEX intravitreal implant is composed of a biodegradable copolymer of polylactic-co-glycolic acid containing micronized dexamethasone [3]. Ozurdex pharmocokinetics enable high concentrations of dexametasone release into retina and vitreous during first 3 months following injection and lower concentrations may still remain up to 6 months [15]. Ocular hypertension and cataract are two major long-term sequelae identified in large, randomized clinical trials. Case reports have shown implant migration, accidental injection into the lens, infection, posterior segment sequelae including vitreomacular traction et.. [16]. In the study, we observed elevated intraocular pressure and cataract formation as complications of intravitreal DEX implant.

Endothelial cell density was decreased at 3 months after intravitreal injection, but there was no statistically significant difference in pleomorphism and polymegatism. Despite increased IOP and decreased ECD at 3 month, there was no statistically significant change in CCT. Intraocular pressure may cause CCT variation by two possible mechanisms. First one is impairment of pump function of corneal endothelium when IOP reaches a critical level above 40 mmHg in human eyes. None of the patients had IOP above 40 mmHg after injection in the study. Another mechanism may be direct effect of elevated IOP on mechanical properties of cornea. Cornea is a nonlinear viscoelastic tissue that presents different mechanical properties under different IOP levels. Goldmann correlated IOP measured by ocular response analyser showed a positive correlation with CCT [17–19]. Endothelial cell function was compromised and corneal transparency was lost when cell density was decreased significantly from average of 3000 cells/mm^2^ to nearly 1000 cells/mm^2^ [20]. Decrease in ECD did not come to a critical level in the study, thus had no effect on CCT. Also increased pleomorphism and polymegatism might have reduced the ability of endothelial cells to hydrate the cornea [20]. Neither pleomorphism nor polymegatism showed statistically significant difference after intravitreal injection and had no effect on mean CCT.

Previous studies have reported different results about effect of intravitreal injections on corneal endothelium. Güzel et al. proposed that endothelial cell density and morphology did not change after intravitreal ranibizumab and bevacizumab injections [21]. Peraz Rico et al. showed that ranibizumab had no harmful effect on corneal endothelium [22]. Although previous immunohistochemistry studies detected mRNA encoding glucocorticoid receptor in corneal endothelium, [7], contraversies exist about effect of dexamethasone implant on corneal endothelium. In a study by İlhan et al., effect of intravitreal dexametasone implant on corneal endothelium has been studied and no statistical difference was found in ECD,CV and Hex during 6 month follow up [9]. Michalska-Małecka et al. reported no statistically significant difference in endothelial cell density of patients with macular edema secondary to BRVO and CRVO at 6 month [23]. Contrary to these studies, in an in vitro study in bovine eyes, corneal endothelial cells were cultured with different concentrations of dexamethasone and cellular apoptosis and necrosis were shown at high concentrations [6].

Unfavorable effect of corticosteroids on regeneration of corneal endothelial cells is well-known [24]. Although relatively rare, DEX implant may migrate to anterior chamber in aphakic eyes, pseudophakic eyes with capsular and zonular defects, vitrectomized eyes and eyes with long axial length. Kang et al. reported 4 patients out of 924 intravitreal DEX injections with 7 episode of anterior chamber migration. All 4 eyes had corneal edema and one eye required corneal transplantation. Corneal edema occured in all patients regardless of injection duration [25]. In a recent peer-reviewed literature, to date 51 cases of DEX implant migration to anterior chamber were reported by Rhimy et al. Corneal endothelial decompansation and edema were present in 74.5% of the patients (38 of 51 patients) and corneal edema was observed if migration occured within 3 weeks. Rhimy at el. hypothesized that mechanism of corneal edema may be secondary to chemical toxicity of implant or from mechanical trauma of the rigid device making direct contact with corneal endothelial surface [26]. In the study, there was no contact of DEX implant and corneal endothelium, therefore we may conclude that chemical toxicity of DEX implant seems to be more probable than mechanical trauma on corneal endothelium.

Small sample size and shorter follow-up time are the limitations of the current study. Also subgroup analysis such as pseudophakic or phakic patients could not be made because of small sample size.

## Conclusions

In the study, dexamethasone implant caused a transient reduction in endothelial cell density but did not change cell morphology in injected eyes. Possible mechanism may be a kind of chemical toxicity from implant. Effect of DEX implant on corneal endothelium should be considered particularly in compromised corneas prior to decision making. Long-term studies with larger number of patients are still needed to clarify the effect of intravitreal dexamethasone implant on corneal endothelial cell layer.

## Abbreviations

BRVO: Branch retinal vein occlussion
CCT: Central corneal thickness
CRVO: Central retinal vein occlusion
CV: Coefficient of variation of cell size
DEX: Dexamethasone
ECD: Endothelial cell density
Hex: Hexagonality
IOP: Intraocular pressure
LOCS: Lens opasification classification system
mRNA: Messenger ribonucleic acids
OCT: Optical coherence tomography
RVO: Retinal vein occlusion
VEGF: Vascular endothelial growth factor
